# High Level of Integration in Integrated Disease Management Leads to Higher Usage in the e-Vita Study: Self-Management of Chronic Obstructive Pulmonary Disease With Web-Based Platforms in a Parallel Cohort Design

**DOI:** 10.2196/jmir.7037

**Published:** 2017-05-31

**Authors:** Esther PWA Talboom-Kamp, Noortje A Verdijk, Marise J Kasteleyn, Lara M Harmans, Irvin JSH Talboom, Mattijs E Numans, Niels H Chavannes

**Affiliations:** ^1^ Public Health and Primary Care Department Leiden University Medical Centre (LUMC) Leiden Netherlands; ^2^ Saltro Diagnostic Centre Utrecht Netherlands; ^3^ Zorgdraad Foundation Oosterbeek Netherlands

**Keywords:** COPD, eHealth, self-management, integrated disease management, self-efficacy, Web-based platform, primary care, chronically ill, blended care

## Abstract

**Background:**

Worldwide, nearly 3 million people die of chronic obstructive pulmonary disease (COPD) every year. Integrated disease management (IDM) improves disease-specific quality of life and exercise capacity for people with COPD, but can also reduce hospital admissions and hospital days. Self-management of COPD through eHealth interventions has shown to be an effective method to improve the quality and efficiency of IDM in several settings, but it remains unknown which factors influence usage of eHealth and change in behavior of patients.

**Objective:**

Our study, e-Vita COPD, compares different levels of integration of Web-based self-management platforms in IDM in three primary care settings. The main aim of this study is to analyze the factors that successfully promote the use of a self-management platform for COPD patients.

**Methods:**

The e-Vita COPD study compares three different approaches to incorporating eHealth via Web-based self-management platforms into IDM of COPD using a parallel cohort design. Three groups integrated the platforms to different levels. In groups 1 (high integration) and 2 (medium integration), randomization was performed to two levels of personal assistance for patients (high and low assistance); in group 3 there was no integration into disease management (none integration). Every visit to the e-Vita and Zorgdraad COPD Web platforms was tracked objectively by collecting log data (sessions and services). At the first log-in, patients completed a baseline questionnaire. Baseline characteristics were automatically extracted from the log files including age, gender, education level, scores on the Clinical COPD Questionnaire (CCQ), dyspnea scale (MRC), and quality of life questionnaire (EQ5D). To predict the use of the platforms, multiple linear regression analyses for the different independent variables were performed: integration in IDM (high, medium, none), personal assistance for the participants (high vs low), educational level, and self-efficacy level (General Self-Efficacy Scale [GSES]). All analyses were adjusted for age and gender.

**Results:**

Of the 702 invited COPD patients, 215 (30.6%) registered to a platform. Of the 82 patients in group 1 (high integration IDM), 36 were in group 1A (personal assistance) and 46 in group 1B (low assistance). Of the 96 patients in group 2 (medium integration IDM), 44 were in group 2A (telephone assistance) and 52 in group 2B (low assistance). A total of 37 patients participated in group 3 (no integration IDM). In all, 107 users (49.8%) visited the platform at least once in the 15-month period. The mean number of sessions differed between the three groups (group 1: mean 10.5, SD 1.3; group 2: mean 8.8, SD 1.4; group 3: mean 3.7, SD 1.8; *P*=.01). The mean number of sessions differed between the high-assistance and low-assistance groups in groups 1 and 2 (high: mean 11.8, SD 1.3; low: mean 6.7, SD 1.4; F1,80=6.55, *P*=.01). High-assistance participants used more services (mean 45.4, SD 6.2) than low-assistance participants (mean 21.2, SD 6.8; F1,80=6.82, *P*=.01). No association was found between educational level and usage and between GSES and usage.

**Conclusions:**

Use of a self-management platform is higher when participants receive adequate personal assistance about how to use the platform. Blended care, where digital health and usual care are integrated, will likely lead to increased use of the online program. Future research should provide additional insights into the preferences of different patient groups.

**Trial Registration:**

Nederlands Trial Register NTR4098; http://www.trialregister.nl/trialreg/admin/rctview.asp?TC=4098 (Archived by WebCite at http://www.webcitation.org/6qO1hqiJ1)

## Introduction

Chronic obstructive pulmonary disease (COPD) is a slowly progressive lung disease and one of the main causes of morbidity and mortality in high-, middle-, and low-income countries [1]. Worldwide, nearly 3 million people die of COPD every year; in 2012, this was equal to approximately 6% of all deaths globally [2,3].

According to current COPD guidelines, symptoms and airflow obstruction should be monitored regularly to guide modification of treatment and for early identification of complications [4,5]. Routine monitoring should contribute to achieving management goals in COPD (ie, to delay disease progression and alleviate its manifestations). The most important primary care objective should be to improve quality of life (QoL) [6].

In the past decade, integrated disease management (IDM) was introduced as a means of improving quality of care. An IDM program for COPD consists of different components of care in which various health care providers cooperate on education, exercise, behavioral therapy, smoking cessation, medication, nutrition advice, and follow-up. The responsibility for the program lies largely with the health care professional, with a modest role for the patient. For people with COPD, IDM not only improves disease-specific QoL and exercise capacity, but it can also reduce hospital admissions and hospital days per person [7].

### Self-Management of Chronic Obstructive Pulmonary Disease

Self-management of COPD has been introduced as an effective method to improve the quality and efficiency of IDM, and to reduce health care costs [8-10]. Interventions to support self-management have shown reductions in hospital admissions and fewer sick days as a result of exacerbations [11,12]. The core components of self-management include education, eliciting personalized goals, psychological coping strategies, formulating strategies to support adherence to treatment, and behavioral change, together with practical and social support [13,14]. Chronically ill patients who have experience with person-centered, high-quality chronic illness care that focuses on patient activation, decision support, goal setting, problem solving, and coordination of care are better self-managers [15]. Self-efficacy explores the emotional functioning and coping skills of an individual and is thought to be a strong predictor of health behavior of COPD patients; the General Self-Efficacy Scale (GSES) tool is a reliable and sensitive measure of self-efficacy for patients with COPD [16].

### eHealth Interventions

Generally, eHealth interventions are effective in stimulating self-management because they allow patients to better cope with their illness at the time/place of their choosing, enabling them to adapt their lifestyle to their condition, while reducing medical staff consultations [17]. The deployment of eHealth apps facilitates accessibility to health care, which enhances patients’ understanding of their disease, their sense of control, and willingness to engage in self-management [18,19]. Although patients’ attitudes and receptiveness toward eHealth apps are promising in persons of a certain age and education level [20-22], large-scale adoption of self-management and eHealth in daily practice lags behind expectations [23].

Previous eHealth studies have revealed the challenges, barriers, and factors that make successful implementation difficult, yet many questions remain unanswered. Moreover, a major challenge of eHealth in care coordination is to make it beneficial and easy to use for health care providers *and* patients, otherwise neither will use it [24]. Also, online self-management support needs to be a fully integrated element of IDM. For example, in a Dutch study on adherence to an online self-management app for patients with COPD or asthma, patients tended to use the online application on a regular basis when the health care provider was involved, whereas patients without assistance used the app only sporadically [25]. For barriers related to clinicians, the eHealth evidence base needs strengthening, whereas for primary care practices a learning process (including staff training) needs to be instituted [26]. In addition, it is necessary to more adequately inform patients about the possibilities and consequences of eHealth [27]. Furthermore, poor user-friendliness of Web-based apps and the lack of “push” factors (eg, automated reminders or messages from health care professionals) are a common cause of low usage or decline in usage of Web-based apps [28]. In any eHealth study, a substantial proportion of users drop out before completion, or stop using the app; thus, attrition is a common problem and should be analyzed to provide data for real-life adoption problems [29]. Studies on the use of online self-management show that attrition tends to start when users “get lost” in the intervention [28,30].

Preconditions for starting eHealth are (1) it must be well organized within usual care (organizational perspective), (2) it should be beneficial and easy to use for patients (human perspective), and (3) the apps have to be technically sound (technical perspective).

### Design of e-Vita COPD

Because low usage of eHealth is an ongoing problem, we designed a multilevel study to investigate the implementation of a self-management Web platform to support patients with COPD in primary care [31]. Because the Web platform provides continuous education and contact with health care professionals, it is expected to help patients better recognize and self-manage exacerbations in an early phase, thereby helping to stabilize their health status.

This study, called “e-Vita COPD,” compares three different approaches to incorporating eHealth via Web-based self-management platforms into the integrated disease management of COPD using a parallel cohort design *.* Also, participants are randomly allocated in two of the cohorts (1 and 2), using the same platform to different levels of personal assistance. All three cohorts incorporated the platforms to different levels; the two levels of personal assistance for patients were a group with high assistance and a group with low assistance. The main aim is to analyze the factors that successfully promote the use of a self-management Web platform for patients with COPD.

From an organizational perspective, our hypothesis is that a self-management Web platform will be better adopted if the platform is an integrated part of IDM, with trained health care professionals who encourage patients to use the platform. From a human perspective, our hypothesis is that a self-management platform will be better adopted by patients if they receive sufficient personal assistance in how to use the platform, and will be better adopted by patients with a higher level of self-efficacy (assessed by GSES) and a higher educational level. From a technical perspective, our hypothesis is that a self-management Web portal will be better adopted if the platform is easy to use and has practical content.

## Methods

### Study Design

For this study, we used the CONSORT-EHEALTH checklist that describes how the intervention should be reported [32].

We designed a quality improvement intervention and chose an implementation study [33]. We designed a method to promote the uptake of our research findings into routine primary health care; with this design, we aimed at studying the influences on health care professionals and patient behavior and at evaluating the process by which these effects are achieved.

**Figure 1:**
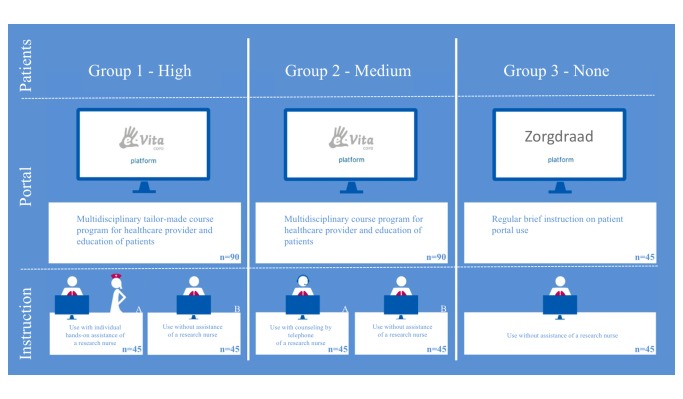
Design of the e-Vita COPD Study. High, medium, and none refer to the level of integration of the web platform into the patient's integrated disease management program. A: high assistance; B: low assistance.

This research combined different study methods to investigate organizational implementation methods and the net benefits of eHealth interventions from a human, organizational, and technical viewpoint. Full methodological details were reported previously [31]. Figure 1 presents an overview of the combined study design with organizational and technical differences.

Included in the study were three different care groups (groups 1-3) and two Web portals (e-Vita and Zorgdraad). In group 1, the online e-Vita platform was offered as a highly integrated part of the COPD IDM with a tailor-made intensive course program on COPD and eHealth for health care providers and patients. Group 2 had a medium level of integration with a standard basic course program for health care providers and patients. The COPD patients in groups 1 and 2 who agreed to use the platform were invited by practice nurses for intake in which they defined a personal health goal together and discussed how and why to use the platform. In group 3, the online platform was offered without integration into disease management; health care providers and patients received instructions from the Web platform itself and received no training on COPD care.

Therefore, group 1 (high) had a high level of integration of the Web platform into their IDM program, group 2 (medium) had a medium level of integration into their IDM program, and group 3 (none) had no integration.

Two different levels of assistance for patients were distinguished within group 1 (high) and 2 (medium): one with a high level of personal assistance and one with a low level of personal assistance. Patients in groups 1 and 2 were randomly subdivided into two groups with high and a low levels of support. Randomization was carried out stratified on the care group (1:1) by computer. In group 1 (high integration-high assistance), a high level of support consisted of two home visits to patients by a research nurse who assisted in the use of the Web portal. In group 2 (medium integration-high assistance), a high level of support consisted of telephone consultation between the patient and a research nurse who explained use of the Web portal. In the low-assistance groups of groups 1 and 2, low-level support consisted of a primary care nurse showing the patient only one time how to use the Web platform, without any follow-up instruction. Patients in group 3 that used the online self-management platform (called “Zorgdraad”) had no active support from health care professionals or research nurses.

Both platforms were provided for the intervention period of 15 months.

### Participants

Three health care groups participated in this study. Patients were eligible if they were diagnosed with COPD according to the Global Initiative for Chronic Obstructive Lung Disease (GOLD) criteria (postbronchodilator forced expiratory volume in first second/forced vital capacity <0.7) in accordance with the COPD Guidelines of Dutch general practitioners (GPs) [34] and they were being treated for COPD in primary care. The study aimed to be inclusive rather than exclusive, to achieve high external validity (applicability to daily practice). Patients were excluded if they were unable to fill in questionnaires, had no access to the Internet, had a terminal illness, were immobile, or were severe substance abusers *.*

#### Recruitment of Patients and Nonparticipation Analysis

We started by recruiting the primary care groups (groups 1-3). Healthcare professionals decided to participate in this study mainly because they wanted to join a project that offered possible health care improvement. In group 1, this was 19 of 170 GPs (11%); in group 2, 29 of 34 GPs (84%); and in group 3, all 27 GPs (100%).

Patients were invited to participate by letter. When they refused to participate, we defined them as nonparticipants. When participants in the e-Vita study logged in and used the Web platform at least once, we defined them as “users.” Patients were defined as “lost to follow-up” if they did not log on to the platform after signing informed consent and if they did not complete the whole intervention period.

### Intervention

The interventions in the three groups consisted of a self-management program including different levels of education for health care professionals, different levels of integration in the COPD care program, and different levels of personal assistance for patients. We used two Web-based platforms (e-Vita in groups 1 and 2, Zorgdraad in group 3) that were very similar, with the same basic features and functionalities. The education, the care programs, and the platforms were specifically designed for COPD patients; their needs and wishes were processed.

The online self-management platform e-Vita is an initiative of the Dutch foundation “Care Within Reach” [35]. The content was created by experts guided by interviews with COPD patients about their thoughts/feelings related to living with COPD and its treatment; the experiences of health care professionals related to the treatment of patients with COPD were also integrated. The main content of the platform consists of insight into personal health data, self-monitoring of health values, education, and a coach for attaining personal goals. The first release of the platform was in January 2014 with an update in May 2015 (which was during the intervention period).

All patients in groups 1 and 2 that used the e-Vita platform had access to a telephonic and digital helpdesk to address any problems. Patients in groups 1 and 2 that used e-Vita received automated online reminders via email from our research team for the self-reported questionnaires and messages.

atients in group 3 used the online self-management platform Zorgdraad (an initiative of the Dutch foundation Zorgdraad and the diagnostic center Saltro). The content was created by experts guided by their experience in treating patients with COPD. The main content of the platform is basic and consists of insight into personal health data, self-monitoring of health values, and education. All patients that used Zorgdraad received automated online reminders via email for the self-reported questionnaires.

### System and Services

When logging on (username plus password) for the first time to e-Vita or Zorgdraad, every user saw a pop-up with a brief explanation about e-Vita or Zorgdraad and the services that could be found on the website. After the pop-up, the user was directed to the home page. From there, users were able to access all functionalities of the platform. The log-on procedure of both platforms is based on Dutch security legislation and guidelines (the Dutch Personal Data Protection Act).

The e-Vita platform (Multimedia Appendices 1 and 2) consisted of the following set of interrelated services, which could be accessed via the home page:

1. An online coach for guidance when working on personal goals and planning of the personal actions.
2. Self-monitoring personal health values and self-reported questionnaires, in which users could register the values they measured for the disease-specific health status Clinical COPD Questionnaire (CCQ) [36], the modified Medical Research Council scale (MRC scale) for dyspnea [37], the GSES [38], sociodemographic characteristics, and EuroQol five-dimensions questionnaire on quality of life (EQ-5D) [39].
3. An education module with text and movies about COPD.
4. Extra information about COPD.
5. Information about the team of health care professionals
6. A module to send and receive messages to the health care professionals.

The Zorgdraad platform (Multimedia Appendix 3) has very basic usability and content, with the following set of interrelated services:

1. Self-monitoring personal health values and self-reported questionnaires, where users can register the same values as in the e-Vita platform.
2. A basic education module with text and movies about COPD.
3. Information about the team of health care professionals.
4. A module to send and receive messages to the health care professionals.

### Outcome Measures

Full details on outcome measures were reported previously [31]. The primary outcome of this study was the usage of the online self-management platform: we defined usage as every visit to e-Vita and Zorgdraad that was tracked objectively by collecting log data in log files. We focused on the log data in the intervention period of 15 months. The usage was divided into sessions and services; a *session* was defined as the total period between logging in and logging out of the system and a *service* was defined as a focused action within the system, as described for both platforms previously. For every action in e-Vita and Zorgdraad (button clicks, page views, and database transactions), the following information was collected by the Web server and added to a log file: (1) the users’ identification number, (2) time and day of the session, (3) the type of actions (services) taken, and (4) optional additional information about the actions (services). For these analyses, it was important to investigate not only the amount of use, but also the user’s patterns.

Baseline characteristics were extracted from the log files of e-Vita and Zorgdraad, including age, gender, education level, and scores on the CCQ (range 0=very good health status to 6=extremely poor health status), MRC scale (range 1-5), EQ-5D, and GSES.

Data were collected that could plausibly be related to the study outcomes. In the analyses, the organizations of GPs (care groups PreventZorg, Zorg op Noord, and Leidsche Rijn Julius Gezondheidscentra), integration in IDM (integrated vs not integrated), and personal assistance for the participants (personal assistance vs no assistance) were used as determinants. Education was self-reported using eight response categories and converted into three levels based on the International Standard Classification of Education (ISCED): high (bachelor, master, doctor), medium (secondary and tertiary education), and low (no formal education, primary education) [40]. Self-efficacy was measured with the GSES in a self-reported 10-item questionnaire. Total scores ranged from 0 to 10.

An attrition curve was drawn with the nonusage attrition; the percentage of users who used the platforms were plotted over time.

### Statistical Methods

For the nonparticipation analysis, differences in age and gender between participants and nonparticipants were compared using a chi-square test and a Mann-Whitney *U* test after normality tests.

Categorical baseline characteristics were reported as numbers and percentages, normally distributed continuous variables as means with standard deviations (SD), and nonnormally distributed variables as medians with interquartile ranges (IQRs). Characteristics between the three groups were explored using chi-square tests and Kruskal-Wallis tests.

To predict the use of the e-Vita and Zorgdraad platforms, multiple linear regression analyses (mean number of sessions/services/mean session time/mean number of services per session) for the different independent variables were performed:

1. Integration in integrated disease management including training by health care professionals by comparing groups 1 (high), 2 (medium), and 3 (none);
2. Personal assistance given to patients by comparing the high-assistance groups of groups 1 (high) and 2 (medium) versus the low-assistance groups of groups 1 (high) and 2 (medium);
3. Patients’ scores on the GSES; and
4. Patients’ educational levels.

We analyzed the main effects because, theoretically, we presumed no interaction between factors. All analyses were adjusted for age and gender.

Attrition was measured by logging and evaluating the percentage of users that used one of the platforms every month during the intervention period plus three months. The area under the curve was calculated for a period of 18 months; after this period, usage dropped to zero for two groups. We used a calculating program to measure the area under the curve using definite integrals.

All analyses were performed with SPSS version 22.0 (IBM Corporation, Armonk, NY, USA).

## Results

**Figure 2:**
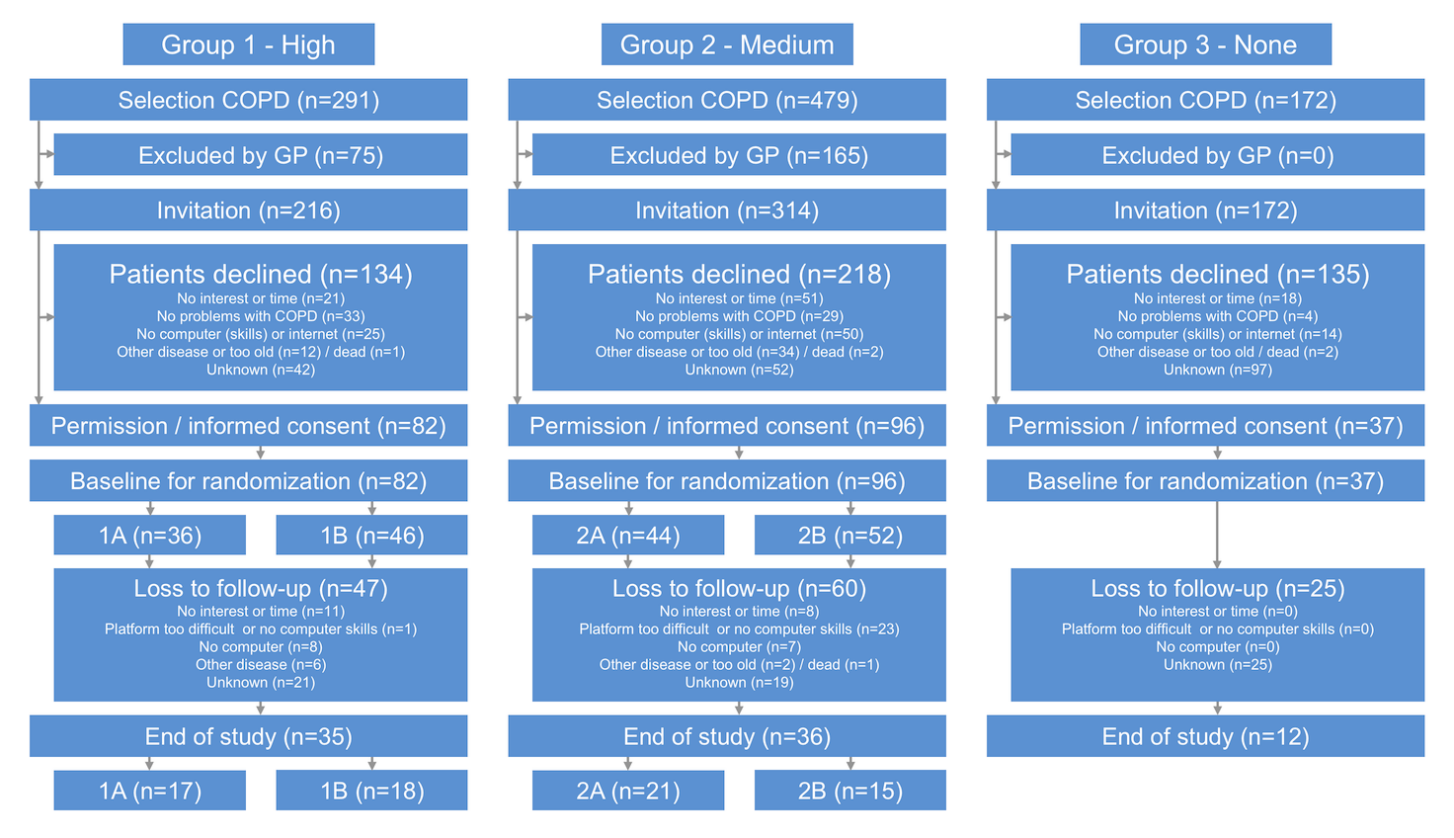
Flowchart of inclusion of participantss in the e-Vita COPD study. High, medium, and none refer to the level of integration of the web platform into the patient's integrated disease management program. A: high assistance; B: low assistance.

In total, 942 diagnosed COPD patients from the medical files of the three care groups were selected to be eligible for the study (Figure 2). The GPs of these care groups excluded 240 COPD patients from participation due to (1) other diseases, (2) treatment in hospital, and (3) probably incompetent to participate in the program. In the end, 702 COPD patients were invited to start with the e-Vita platform; of these, 215 (30.6%) agreed to register and provided informed consent. Reasons for declining to participate are presented in Figure 2.

Of the 215 COPD patients included at baseline, 82 were in group 1 (high), 96 in group 2 (medium), and 37 in group 3 (none).

After randomization, 36 patients in group 1 were allocated to high-assistance group, 46 to low-assistance group; in group 2 44 patients were randomized to high-assistance group, and 52 to low-assistance group.

**Figure 3:**
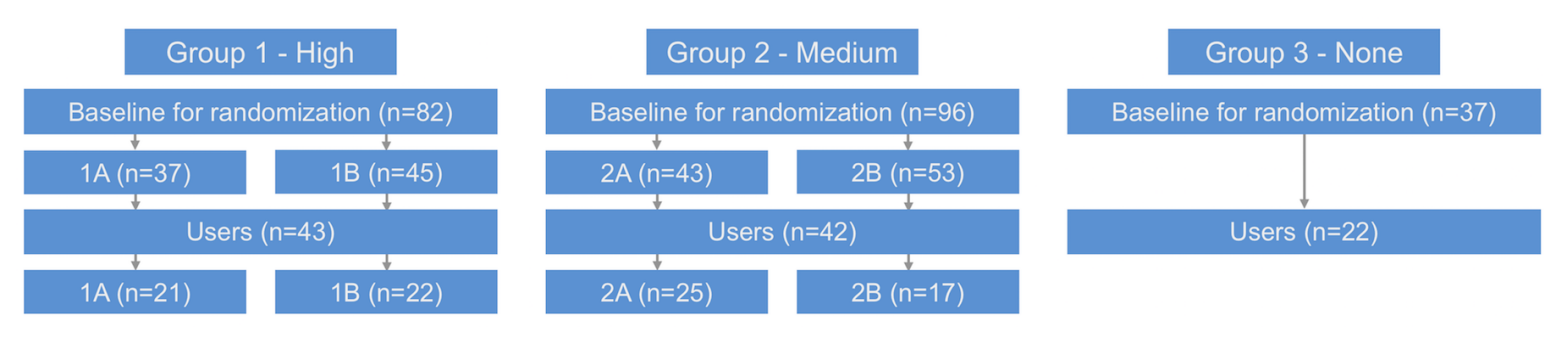
Flowchart of the platform users of the e-Vita COPD study. High, medium, and none refer to the level of integration of the web platform into the patient's integrated disease management program. A: high assistance; B: low assistance.

The total number of patients lost to follow-up was 132. Figure 2 shows the reasons for drop out in groups 1 and 2; patients in group 3 were not asked for their reasons. Of the 215 participants, 107 (49.8%) patients were identified as platform users: 43 in group 1, 42 in group 2, and 22 users in group 3 (Figure 3).

**Table 1:** Characteristics and comparison of participants and nonparticipants of the e-Vita study.

Nonparticipants/participants		Group 1 (high)	Group 2 (medium)	Group 3 (none)	Total
**Nonparticipants**					
	n	209	383	135	727
	Age (years), median (IQR)	69.3 (61.1-77.5)	69.8 (61.6-78.5)	66.3 (60.3-74.4)	68.5 (61.2-77.9)
	Males, n (%)	108 (51.7)	167 (43.6)	61 (46.2)	336 (46.2)
**Participants^a^**					
	n	82	96	37	215
	Age (years), median (IQR)	66.3 (61.1-75.7)	67.3 (62.6-76.6)	64.1 (61.5-69.2)	66.6 (61.4-74.7)
	Males, n (%)	51 (62.2)	41 (42.7)	20 (54.1)	112 (52.1)

^a^Baseline for randomization.

**Table 2:** Baseline demographic and clinical characteristics of the patients with COPD included in the e-Vita study.

Participants		Group 1 (high)		Group 2 (medium)		Group 3 (none)	Total
		High	Low	High	Low		
n		36	46	44	52	37	215
Age (years), (IQR)		66.3 (61.0-79.2)	65.6 (61.3-73.4)	68.7 (64.0-78.3)	66.8 (60.3-75.1)	64.1 (61.5-69.2)	66.6 (61.4-74.7)
Male, n (%)		19 (52.8)	32 (69.6)	17 (38.6)	24 (46.2)	20 (54.1)	112 (52.1)
**Education level, n (%)**							
	Low	4 (28.6)	8 (38.1)	5 (22.7)	8 (42.1)	7 (53.8)	32 (36.0)
	Medium	7 (50.0)	8 (38.1)	11 (50.0)	8 (42.1)	4 (30.8)	38 (42.7)
	High	3 (21.4)	5 (23.8)	6 (27.3)	3 (15.8)	2 (15.8)	19 (21.3)
**Questionnaire, median (IQR)**							
	CCQ	1.0 (0.6-1.9)	1.2 (0.8-1.6)	1.3 (0.9-2.1)	1.4 (1.1-2.1)	1.3 (0.6-1.8)	1.2 (0.8-1.9)
	mMRC scale	1.0 (1.0-3.0)	1.0 (1.0-2.0)	2.0 (1.0-3.0)	2.0 (1.0-2.0)	1.0 (1.0-1.0)	1.0 (1.0-2.0)
	GSES	3.4 (3.1-3.7)	3.3 (3.0-3.8)	3.3 (2.8-3.5)	3.3 (3.1-3.7)	3.4 (3.3-3.7)	3.3 (3.0-3.7)
	EQ-5D	0.85 (0.7-1.0)	0.89 (0.81-1.0)	0.85 (0.72-1.0)	0.84 (0.71-1.0)	0.9 (0.84-1.0)	0.86 (0.78-1.0)

### Nonparticipation Analysis

The age and gender of participants and nonparticipants are presented in Table 1. Participants and nonparticipants did not differ with regard to gender (52.1%, 112/215 male vs 46.2%, 336/727 male, *P*=.13) or age (median 66.6, IQR 61.4-74.7 vs median 68.5, IQR 61.2-77.9 years, *P*=.20). Because only a few nonparticipants filled in a questionnaire on CCQ, the mean CCQ could not be determined for nonparticipants.

### Baseline Characteristics of Patients

Table 2 presents the baseline demographic and clinical characteristics of the included COPD population (median age 66.6 years; 52.1% was male). These patients had mildly symptomatic COPD which is reflected by a median MRC scale of 1.0 and a median CCQ of 1.2. Of all participants, 89 of 215 (41.4%) filled in the online questionnaire for education level; most participants had a middle education level (42.7%). The median GSES was 3.3 and the median EQ-5D 0.86. The characteristics age (χ^2^_2_=5.4, *P*=.07), education level (χ^2^_4_=2.2, *P*=.70), GSES (χ^2^_2_=1.74, *P*=.42), and EQ-5D (χ^2^_2_=2.4, *P*=.28) were similar in the three groups. There was a difference in the characteristics gender (χ^2^_2_=6.8, *P*=.03), with more male patients in group 1; and a difference in CCQ (χ^2^_2_=6.5, *P*=.04) and MRC scale (χ^2^_2_=11.3, *P*=.003) with a higher CCQ and MRC scale in group 2.

### Use of the Online e-Vita and Zorgdraad Platforms

Table 3 presents the 15-month usage pattern by patients using the log files of e-Vita and Zorgdraad. In total, 107 users visited the platform at least once in the 15-month period. The helpdesk received 101 calls; most questions concerned problems with the log-on procedure.

**Table 3:** Usage patterns for groups 1, 2, and 3.

Usage^a^	Group 1 (high)	Group 2 (medium)	Group 3 (none)	Total	*P*	*F*_2,100_
n	43	42	22	107		
Sessions, mean (SD)	10.5 (1.3)	8.8 (1.4)	3.7 (1.8)	8.2 (8.7)	.01	4.68
Session time (minutes), mean (SD)	3.5 (0.7)	4.8 (0.7)	6.7 (0.9)	4.8 (4.3)	.03	3.83
Total services per user, mean (SD)	45.2 (6.1)	27.9 (6.2)	6.7 (8.3)	28.8 (41.0)	.001	7.18
Total services per session per user, mean (SD)	3.9 (0.4)	4.1 (0.4)	2.1 (0.6)	3.6 (2.8)	.02	3.97

^a^ Adjusted for age and gender.

**Figure 4:**
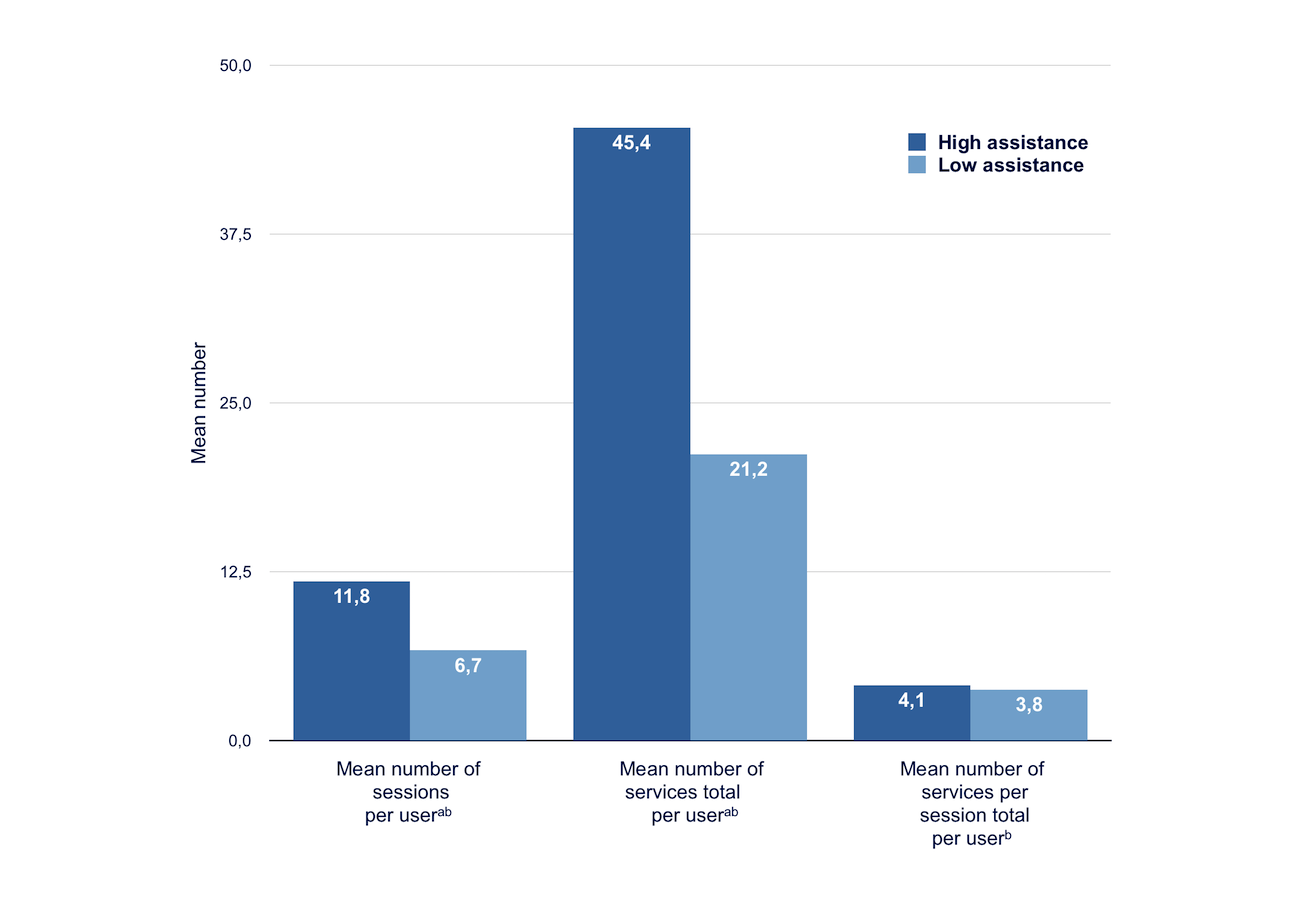
Usage patterns for high and low levels of assistance. a: mean number significantly higher in high assistance group b: adjusted for age and gender.

In the 15-month intervention period, the total number of sessions for the e-Vita platform was 830 (9.8 per user) and for the Zorgdraad platform 78 (3.5 per user). The mean number of sessions differed between the three groups ((group 1: mean 10.5, SD 1.3; group 2: mean 8.8, SD 1.4; group 3: mean 3.7, SD 1.8; *P*=.01) (Table 3). In groups 2 and 3, the mean session time was higher than in group 1; the difference between the three groups was significant (*P*=.03). In groups 1 and 2, the mean number of services in total was higher than in group 3 (group 1: mean 45.2, SD 6.1; group 2: mean 27.9, SD 6.2; group 3: mean 6.7, SD 8.3; *P*=.001) and the number of services per session in groups 1 and 2 was higher than in group 3 (group 1: mean 3.9, SD 0.4; group 2: mean 4.1, SD 0.4; group 3: mean 2.1, SD 0.6; *P*=.02).

Figure 4 shows use of the e-Vita for the two groups with high and low assistance; higher usage of the platform was related to a higher level of personal assistance.

The mean number of sessions differed between the high-assistance groups and the low-assistance groups in groups 1 and 2 (high assistance: mean 11.8, SD 1.3; low assistance: mean 6.7, SD 1.4; *F*_1,80_=6.55, *P*=.01). Participants in the high-assistance groups used more services (mean 45.4, SD 6.2) than participants in the low-assistance groups (mean 21.2, SD 6.8; *F*_1,80_=6.82, *P*=.01). In the high-assistance groups, the mean number of services per session did not differ from the low-assistance groups (mean 4.1, SD 0.4 vs mean 3.8, SD 0.5; *F*_1,80_=0.36, *P*=.55).

**Figure 5:**
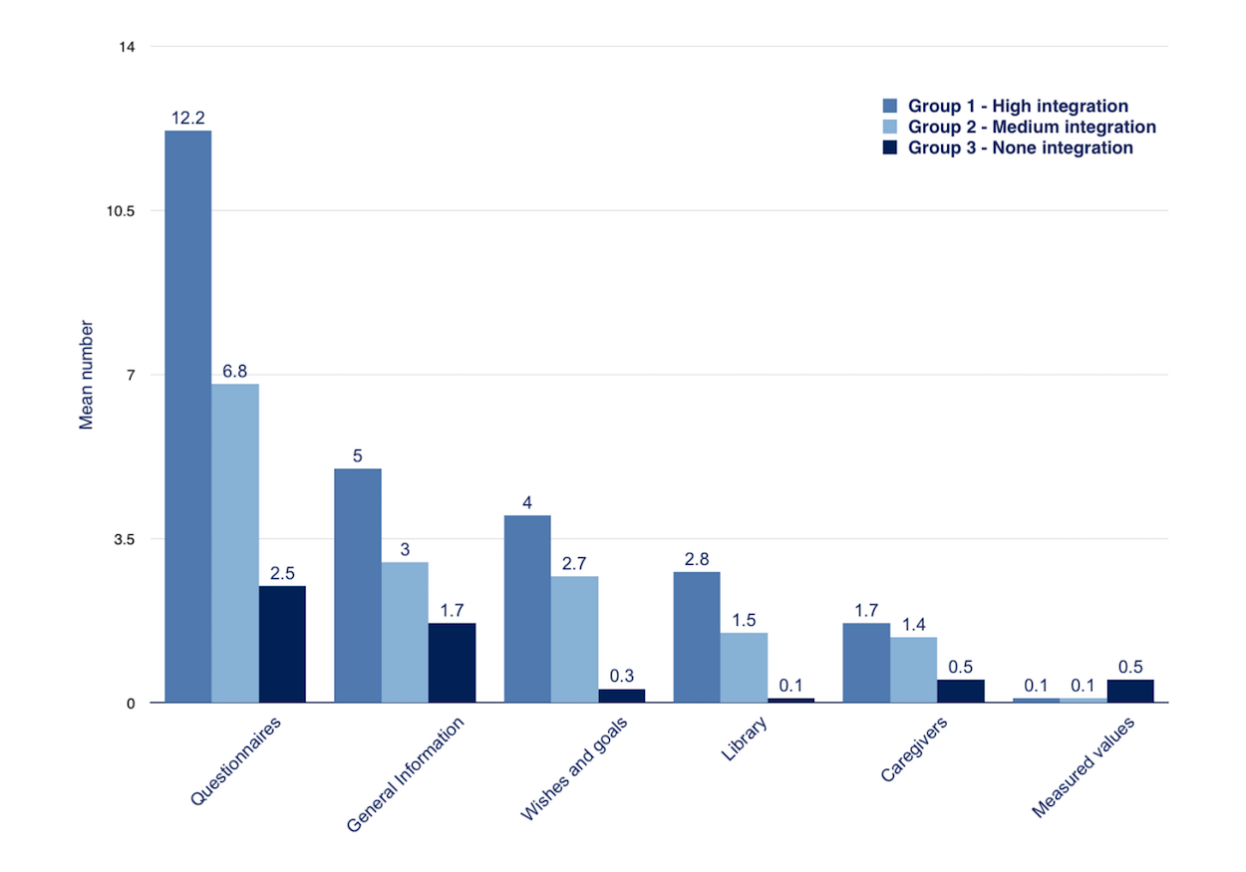
Usage patterns of the mean number of services per user in each group.

An overview of the online platform services visited during the intervention period is provided in Figure 5; for every service, the mean number per user is depicted. The log files revealed that all services were mainly used by group 1. Furthermore, it revealed that the e-Vita and Zorgdraad Web platforms were predominantly used for online questionnaires, general information, and depicting wishes/goals related to their lifestyle, and to a lesser extent for online education, visiting the library, and looking for information about their health care professionals. Log files also showed that there was almost no interest in the measurement values CCQ and MRC scale. The email feature and an explanation of the test results of e-Vita were used to a moderate extent.

**Table 4:** Educational level and the General Self-Efficacy Scale (GSES) as predictors for Web platform usage.

Education/GSES			Unadjusted for age and gender				Adjusted for age and gender		
			B (95% CI)		*P*		B (95% CI)		*P*
**Education**					.20				.15
	Low	–4.40 (–9.51 to 0.71)				–5.30 (–10.86 to 0.26)			
	Medium	–1.27 (–5.90 to 3.39)				–1.85 (–6.61 to 2.90)			
	High	0.00				0.00			
GSES			0.23 (–4.85 to 5.31)		.93		0.67 (–4.43 to 5.78)		.80

**Figure 6:**
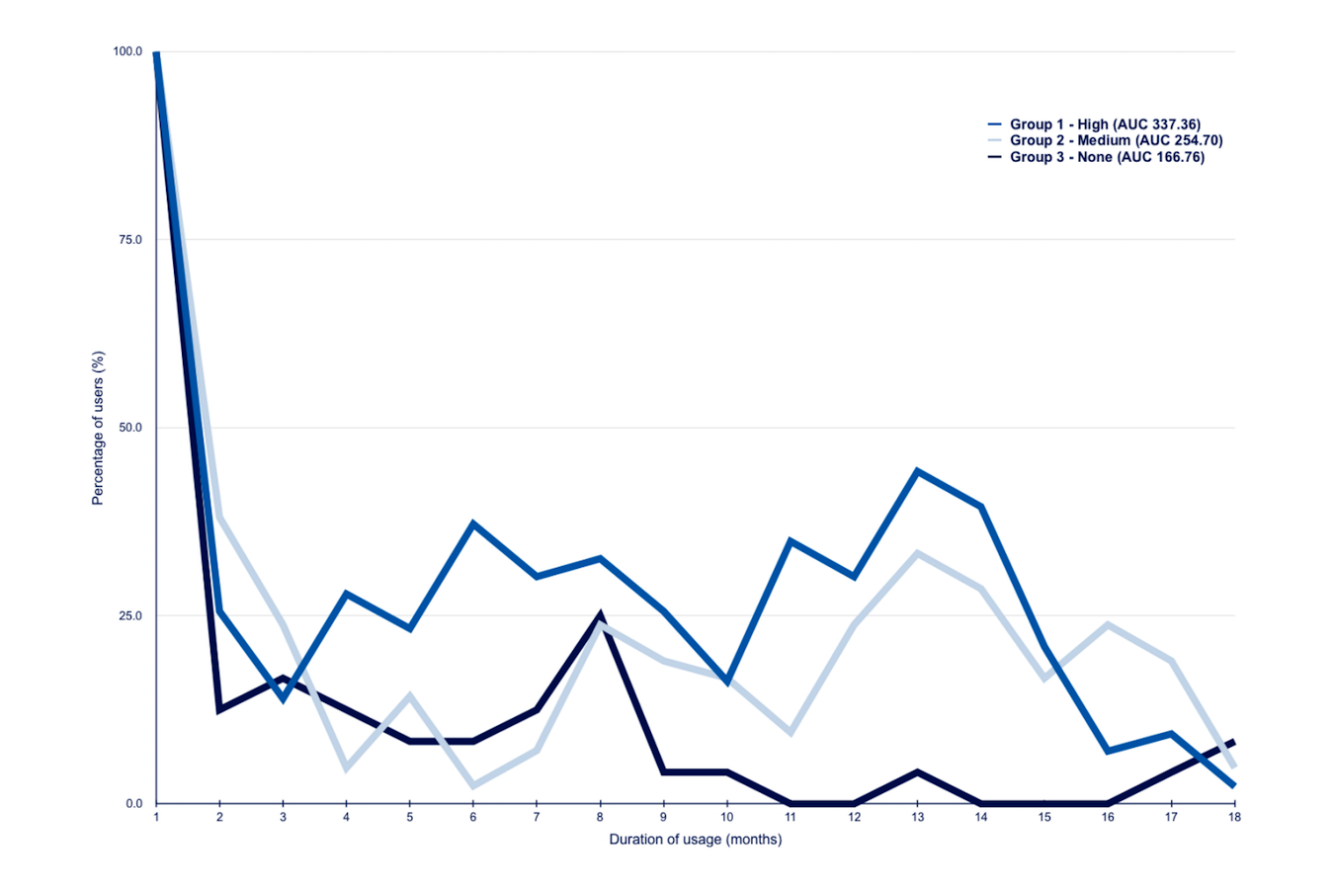
Attrition curve of group 1, 2 and 3.

### Educational Level and the Generalized Self-Efficacy Scale

The association between the educational level and scores on the GSES and the mean number of sessions are presented in Table 4. Educational level was not associated with the number of sessions (*P*=.15). No association was found between the GSES and the mean number of sessions (*P*=.79).

### Attrition

The log files revealed that a substantial proportion of the users did not continuously use the platforms before completion of the study. Figure 6 shows the patterns of use of the Web platforms in groups 1 to 3 during the intervention period, with the percentage of users on the y-axis, starting with 100% of the users, and the duration of usage on the x-axis. The area under the curve (AUC) until the 18th month for attrition in group 1 was 337.36, 254.70 in group 2, and 166.76 in group 3.

## Discussion

### Main Results

In this study, usage of the COPD self-management Web-based platform is higher when the platform is an integrated part of IDM with trained health care professionals who encourage patients to use the platform. Furthermore, usage of the e-Vita COPD platform is higher when patients receive more personal assistance in learning how to use the platform. Usage of the self-management Web-based platform e-Vita (high and medium level of integration in IDM) is higher than that of Zorgdraad (no integration in IDM).

### Interpretation and Findings

Despite high expectations and numerous initiatives in the area of eHealth, implementation and use of eHealth apps are not yet common practice. Our primary aim was to analyze the factors that successfully promote the usage of two self-management Web platforms for COPD patients. We compared different organizational implementation methods. An implementation setup with greater personal support is expected to increase the use of an online program.

Our findings highlight the importance of integrating a Web-based platform into IDM; usage of the self-management Web platform is higher and more varied when the platform is an integrated part of IDM with appropriate personal coaching for patients. Patients in care groups with a high level of integration of the platform in IDM showed a higher number of sessions and a larger amount of visited services with more variation. Patients that received personal assistance also showed higher usage of the platform. Similar results were found in a study on COPD and asthma patients; the online app was used on a more regular basis with higher involvement of the health care provider and more assistance of the patients [25]. The e-Vita study on patients with diabetes mellitus showed minimal impact of implementing a personal health record including self-management support in primary diabetes care; recommendations were made to use additional strategies for patient motivation and engagement of professionals for a successful adoption of Web-based platforms [41,42].

In this study, we implemented extensive professional training of health care professionals on IDM and self-management supported by platforms; we also offered personal assistance for the users to guide them through the platform as well as push factors (automated reminders or messages by health care professionals). Both strategies are essential elements to influence the use of platforms.

The self-efficacy of users (GSES) was not a predictor for use of the platforms. The construct of perceived self-efficacy reflects an optimistic self-belief [43]; a correlation can be understood based on the belief that one can change behavior, perform a novel or difficult task (eg, using a platform), or cope with adversity with a higher GSES [44,45]. In a healthy Dutch population, the mean GSES is reported to be approximately 3.1 [38,46]; in our study population, the median GSES was 3.3. Educational level was not a predictor of use of the e-Vita COPD platform. These predictors might be useful for future studies on and the development of platforms.

The EQ-5D values reflect the relative desirability of health states on a scale in which 1 refers to full health and 0 refers to death. In our study population, the median EQ-5D was 0.86 compared with 0.87 in a healthy Dutch population [47].

In the three groups, there was no bias regarding baseline age, gender, education level, CCQ, MRC scale, GSES, and EQ-5D.

Analysis of attrition provided insight into the decrease in usage (eg, after 1 month, 10%-45% of the participants were actively using the platform). The periodic steep rise in the percentage of users might be explained by the email reminders sent by the platform to fill in the questionnaires; all users received continuous reminders during the intervention period. In group 3, all users received urgent and repeated requests to fill in questionnaires at the end of the intervention period, which probably explains the steep rise in the percentage of users at the end of the study. The attrition curve depicts the “push factors” that are required to remind participants to use the platform. This “law of attrition” (the phenomenon of participants stopping usage) is a common finding in eHealth evaluations and one of the fundamental and methodological challenges in the evaluation of eHealth apps [29].

During this study, there were several lessons learned by the research team. First, it took a lot of effort to motivate health care professionals to work together with patients on self-management platforms; we experienced differences in communication skills among the health care professionals working with patients in a more modern, less hierarchic way. When patients started using the platform, it took great effort to stimulate the usage with several reminders, even though we established a high amount of attrition probably due to low usability of the platforms and logging problems.

### Strengths and Limitations

This e-Vita COPD study has several strengths. To our knowledge, it is the first to combine different study designs thereby enabling simultaneous investigation of clinical effects, as well as the effects of different organizational implementation methods. Randomization was carried out for the level of assistance for patients. This study also adds evidence to the existing body of knowledge; this is important because local political and financial factors have a major impact on successful integration of eHealth in daily practice [48].

This study also has limitations. Although well-conducted randomized trials provide the most reliable evidence on the effectiveness of interventions, they are not feasible for our setting of an implementation design with organizational changes in a real-life health care system within three different care groups with different demands.

From a technical perspective, development of the Web-based platforms was difficult due to a lack of broad experience in the field. We used two different platforms with the same basic principles and functionalities. The platforms were new and the usability was not tested thoroughly before starting the study. The platform technique and the decisions made during the design phase were beyond the influence of our research group, but have affected our outcomes. From a human perspective, self-management skills imply behavioral changes. Behavioral changes require time, whereas the study period was restricted to 15 months. Furthermore, patients in a primary care setting have a low burden of disease (in this study, the mean CCQ score was 1.2) and motivation to use the platform might be negatively influenced by this fact. In respiratory medicine, there is a lack of research on patients with mild to moderate COPD despite that more than 80% of COPD patients suffer from this stage of disease and are often treated in primary care [49]. From an organizational perspective, other projects in primary care cooperatives can influence the speed and thoroughness of the implementation of our Web portal. Finally, this study also has typical limitations found in eHealth trials. The loss to follow-up is high, as in all eHealth studies. Because general practices, as well as patients, were free to volunteer, bias might have occurred in our research groups. Users were self-selected and were presumably motivated to use the Web-based platform as would be expected in a real-life setting. The patients that were invited by GPs/nurses to participate in the study might differ from other patient groups. Furthermore, GPs excluded 26% of the COPD patients from this study. Of the 702 eligible patients, 215 (30.6%) were willing to participate and provided informed consent, and 132 (61.4%) of the participants dropped out during follow-up. Even though nonparticipants did not differ in age and gender from participants, caution is required when generalizing these results to general practice as a whole. However, the practical applicability of our results for other primary care groups is positive (ie, the study provides practical insight into successful implementation of patient platforms). Nevertheless, primary care organizations should take into account the different aspects of good organization of blended care and good quality of implementation.

More studies are needed (preferably with larger sample groups and among the nonusers) to gain more insight into the preferences of various patient groups. The substantial workload generated by integrating a Web-based platform in IDM emphasizes the importance of piloting and assessing workforce implications for primary care groups during the planning and implementation phase. These results provide additional insight into the organizational aspects of the implementation of platforms, including the need to assist patients in the use of Web-based platforms integrated in IDM.

### Conclusions

Use of a self-management Web-based platform is higher when the platform is an integrated part of IDM, with trained health care professionals encouraging patients to use the platform.

Use of a self-management platform is higher when participants receive adequate personal assistance about how to use the platform.

The self-management Web-based platform e-Vita had higher usage than Zorgdraad, which is probably due to the superior organizational conditions of integrated care and because e-Vita is, technically, better customized for use. An implementation setup with blended care through integration of the online platform in IDM, together with greater personal support of the users, will likely lead to increased use of the online program. Future research should provide additional insights into the preferences of different patient groups.

## Appendix

Multimedia Appendix 1 Homepage e-Vita.

Multimedia Appendix 2 Getting started with the e-Vita platform - https://www.youtube.com/watch?v=h3MDKgPCUJg.

Multimedia Appendix 3 Homepage Zorgdraad.

## Abbreviations

CCQ: Clinical COPD Questionnaire
COPD: chronic obstructive pulmonary disease
EQ-5D: EuroQol 5D
GP: general practitioner
GSES: General Self-Efficacy Scale
IDM: integrated disease management
MRC scale: modified Medical Research Council dyspnea scale
QoL: quality of life
