# Supplementary figures and images for: High Level of Integration in Integrated Disease Management Leads to Higher Usage in the e-Vita Study: Self-Management of Chronic Obstructive Pulmonary Disease With Web-Based Platforms in a Parallel Cohort Design
Source: J Med Internet Res. 2017 May 31;19(5):e185. doi: 10.2196/jmir.7037 (PMC5471344; doi:10.2196/jmir.7037)

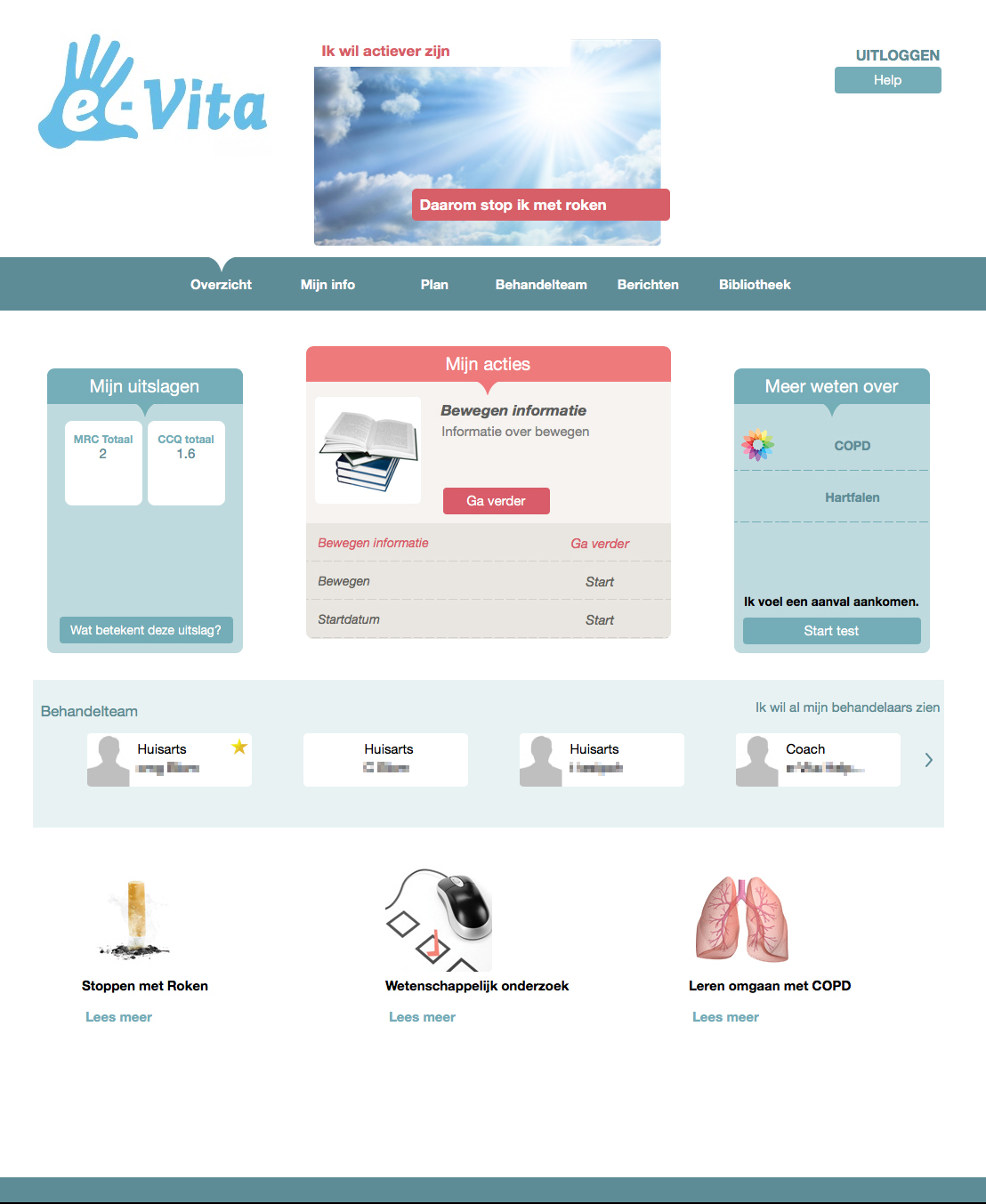

Supplement: Multimedia Appendix 1 [file jmir_v19i5e185_app1.jpg]

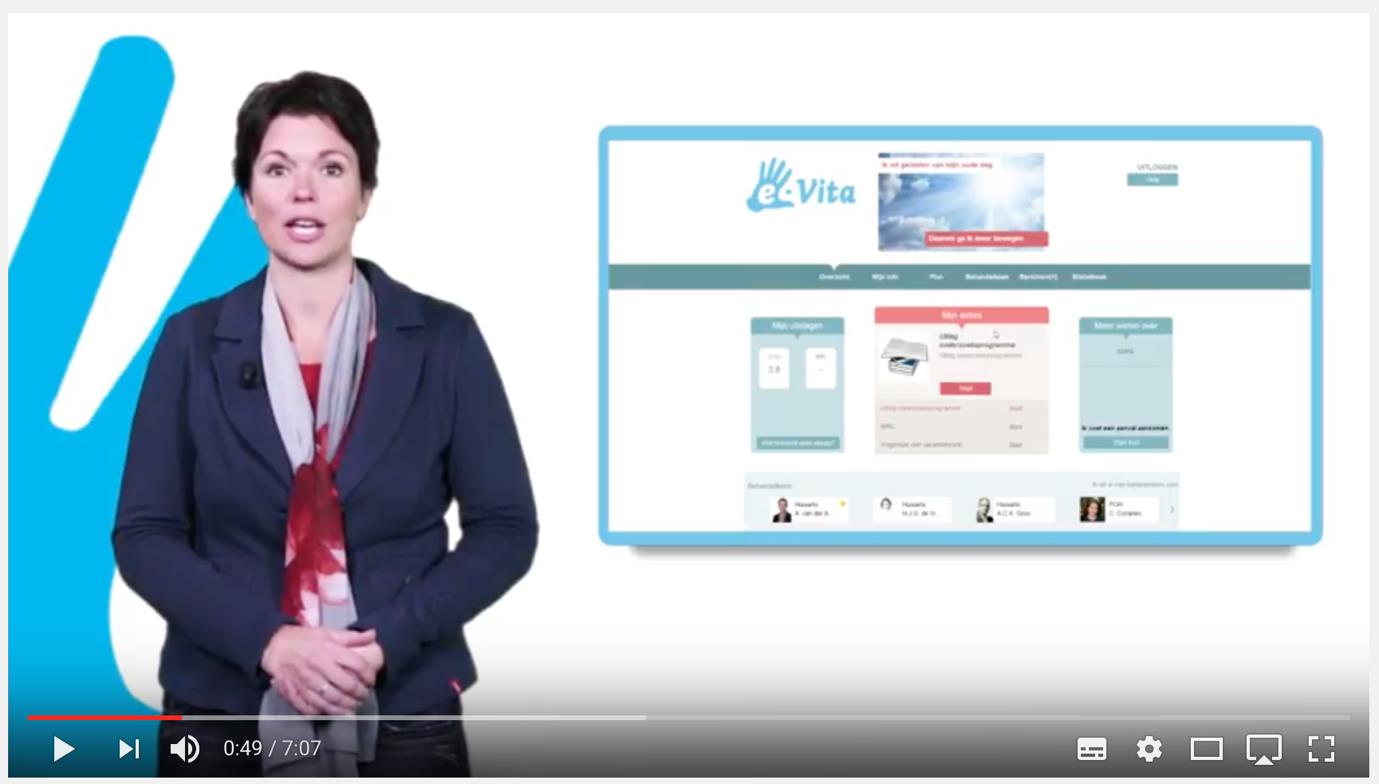

Supplement: Multimedia Appendix 2 [file jmir_v19i5e185_app2.jpg]

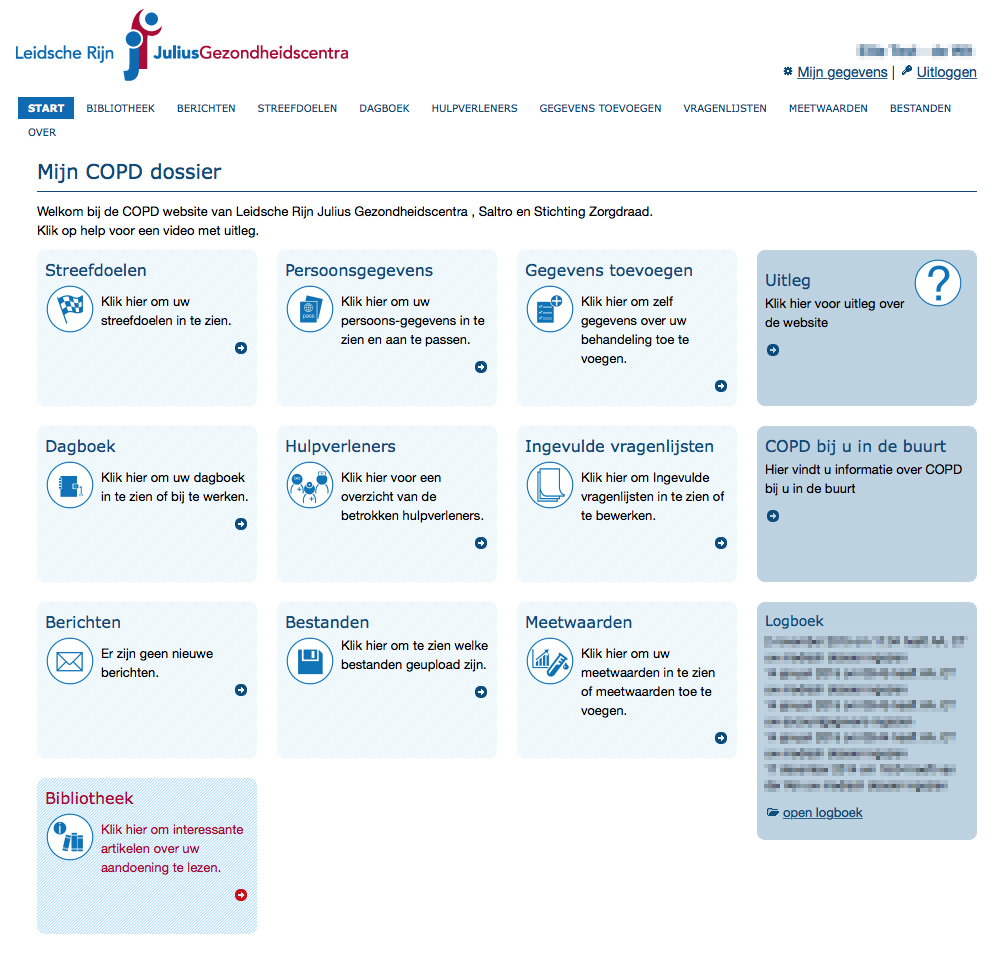

Supplement: Multimedia Appendix 3 [file jmir_v19i5e185_app3.jpg]
